# The First Report of a Non-Canonical Telomeric Motif in Neuroptera: (TTGGG)*n* in Chromosomes of *Nineta flava* (Scopoli, 1763), Chrysopidae

**DOI:** 10.3390/genes16101201

**Published:** 2025-10-14

**Authors:** Desislava Stoianova, Snejana Grozeva

**Affiliations:** 1 Institute of Biodiversity and Ecosystem Research, Bulgarian Academy of Sciences, 1000 Sofia, Bulgaria; sngrov@gmail.com; 2 National Museum of Natural History, Bulgarian Academy of Sciences, 1000 Sofia, Bulgaria

**Keywords:** chromosome structure, Chrysopinae, genome assembly, Nothochrysinae, (TTAGG)*n*, insect cytogenetics

## Abstract

**Background:** Telomeres are nucleoprotein complexes that maintain chromosome integrity in eukaryotes. In insects, the canonical telomeric repeat (TTAGG)*n* is considered ancestral, though alternative motifs exist across various orders. Neuroptera, comprising about 5800 species, remains understudied regarding telomeric sequences, with data available for only seven species across three families. Previous studies reported the absence of (TTAGG)*n* in Chrysopidae species, contrasting with its presence in other Neuroptera families. This study aimed to identify and characterize telomeric motifs in Chrysopidae using chromosome-level genome assemblies and search for retrotransposon insertions. **Methods:** We analyzed chromosome-level genome assemblies from four Chrysopidae species: three Chrysopinae—*Chrysoperla carnea* (Stephens, 1836), *Chrysopa pallens* (Rambur, 1838), and *Nineta flava* (Scopoli, 1763); and one Nothochrysinae—*Nothochrysa capitata* (Fabricius, 1793). Terminal sequences of chromosome pseudomolecules were examined using Geneious Prime^®^, applying five specific criteria for optimal telomeric sequence identification. We searched for SART and TRAS retrotransposons using the graphical sequence panel in GenBank. **Results:** We identified (TTGGG)*n* as the telomeric motif in *N. flava*, representing the first report of this pentanucleotide repeat in telomeres of Neuroptera. Arrays ranged from 228 to 8005 bp across seven terminal locations in five chromosome pseudomolecules. In *N. capitata*, we detected (TTAGG)*n* arrays (2316–3808 bp) at four terminal locations. No telomeric motifs meeting all criteria were found in *C. carnea* and *C. pallens*. No SART/TRAS retrotransposons were detected in any species. **Conclusions:** This study reveals previously unknown telomeric diversity within Chrysopidae, with both canonical (TTAGG)*n* and novel (TTGGG)*n* motifs present. The discovery of (TTGGG)*n* in Neuroptera expands known telomeric sequence diversity in this order.

## 1. Introduction

Telomeres are nucleoprotein complexes at chromosome ends of eukaryotеs that maintain chromosome end integrity and contribute to genome stability [1,2,3,4]. In most eukaryotes, telomeric DNA consists of short G-rich tandem repeats—typically 5–8 bp [5]—bound by telomere-binding proteins that assemble a protective end cap [2,4]. In vertebrates, the hexameric repeat (TTAGGG)*n* predominates [6]. In insects, the telomeric repeat (TTAGG)*n* forms tandem arrays at chromosome ends [7,8,9]. This motif is considered ancestral for Insecta because it was found in most major insect orders and is conserved in basal apterous insect orders, and its phylogenetic distribution pattern supports it being an ancestral motif that was lost repeatedly during insect evolution [7]. The motif is widespread but not universal, since independent losses or replacements occur in multiple lineages (e.g., Diptera, Lepidoptera, many Heteroptera; diverse motifs in Hymenoptera) [10,11]. Many insects carry alternative repeats 1–11 bp long. Some Diptera families, such as Syrphidae and Tachinidae, exhibit much longer terminal repeats, up to 381 bp [12,13,14,15,16,17,18,19,20]. However, data on most insect groups is very scarce compared to their species’ richness. One such group is Neuroptera, a holometabolous insect order comprising about 5800 described species in 15 families [21]. Telomeres have been studied only in seven species belonging to three Neuroptera families: Myrmeleontidae, Ascalaphidae, and Chrysopidae [7,22,23,24]. The presence of the (TTAGG)*n* telomeric motif has been reported for all the studied species in two of these families—Myrmeleontidae, with three analyzed species [23], and Ascalaphidae, with two analyzed species [22,23]. In contrast, for both studied species of Chrysopidae (green lacewings)—*Chrysoperla carnea* (Stephens, 1836) [7] and *Ceraeochrysa claveri* (Navás, 1911) [24]—the absence of (TTAGG)*n* has been reported. The former species has been studied by Southern blotting [7], while the latter—by fluorescence in situ hybridization (FISH) [24]. Genome data for the Chrysopidae species *C. carnea* has been analyzed also using Telomeric Repeats Identification Pipeline (TRIP), a bioinformatics tool that identifies telomeric repeat motifs from Illumina short-read sequencing data by extracting and profiling 2–25 bp tandem repeats, computing 19 summary statistics including repeat-containing read counts and total repeat lengths, and ranking candidates primarily based on abundance metrics. TRIP designates a final telomeric repeat motif when a candidate shows a threshold higher abundance than other repeat units, or when this threshold is not met but additional evidence from literature or high-quality genome assemblies supports the designation. However, this pipeline did not identify any short tandem repeat telomeric motifs in the analyzed Neuroptera genome data [11].

Several noncanonical telomeric motifs have been identified in multiple insect orders, often from analyses of chromosome-level genome assemblies [25,26,27]. In addition, analyses of genome data, particularly in insects [28], have revealed insertions of the telomere-targeting non-LTR retrotransposons SART and TRAS into telomeric tandem-repeat regions. In such cases, telomeres comprise both telomerase-derived repeats and retrotransposon arrays [25].

However, chromosome-level assembly analyses have not been applied to Neuroptera. To our knowledge, neither noncanonical telomeric motifs nor insertions of the telomere-targeting non-LTR retrotransposons SART and TRAS have been reported in Chrysopidae.

We aimed to identify and characterize candidate telomeric motifs in Chrysopidae using chromosome-level genome assemblies, and in addition, we searched for SART/TRAS insertions.

## 2. Materials and Methods

Chromosome-level genome assemblies were available in GenBank for four Chrysopidae species: three Chrysopinae (*C. carnea*, *Chrysopa pallens* (Rambur, 1838), and *Nineta flava* (Scopoli, 1763)) and one Nothochrysinae (*Nothochrysa capitata* (Fabricius, 1793)), with accession numbers GCA_905475395.1, GCA_020423425.1, GCA_963920215.1, and GCA_965240235.1, respectively. We downloaded these publicly available assemblies, all of which had been generated using long-read sequencing technologies essential for accurately resolving repetitive telomeric regions that are typically problematic for short-read sequencing approaches. Specifically, *N. flava* and *N. capitata* had been assembled using PacBio and Arima2 technologies; *C. carnea* using PacBio, Illumina, and Arima technologies; and *C. pallens*—using PacBio Sequel technology.

We applied an approach similar to that in [25,26,27], loading each assembly into Geneious Prime^®^ 2025.0.3 and examining the terminal sequences through side-by-side comparison of the terminal regions of the chromosome pseudomolecules within each assembly.

Additionally, we applied five specific requirements that candidate short motifs must fulfill to qualify as optimal telomeric sequence candidates [25,26]: (1) the motifs must occupy strictly terminal locations; (2) their size should span a minimum of 300–400 bp (see [29]); (3) across a species, any candidate telomeric motif must display identical sequences in all chromosome pseudomolecules where detected; (4) within each analyzed chromosome pseudomolecule end, alternative motifs should be either absent or extremely uncommon; and (5) telomeric motifs positioned at opposite chromosome pseudomolecules’ ends must exhibit reverse-complementary organization and directionality.

To identify SART and TRAS elements in the chromosome-level genome assembly chromosome pseudomolecules in species with canonical motifs, we employed the method outlined in [25]. In this approach, the sequences “AAAAAAAAAACCTAACCTAA/TTAGGTTAGGTTTTTTTTT” and “CCTAACCTAACCTTTTTTTTTT/AAAAAAAAAAGGTTAGGTTAGG” served as search templates for detecting retrotransposons belonging to the TRAS and SART families, respectively. The search was carried out using the “Find” tool in the graphical sequence panel (format ‘Graphics’) implemented in GenBank; the tool was used to find exact matches of the query sequences. For species exhibiting alternative motifs, the search templates were adjusted to correspond with the alternative sequence identified in each respective species.

## 3. Results

At the chromosome pseudomolecules’ ends of *C. carnea* and *C. pallens*, we failed to detect motifs that met all five selection criteria (listed in the Section 2) for telomeric sequences.

The best candidate for the telomeric motif in *N. flava* was the pentanucleotide (TTGGG)*n*. It occurred at the ends in five of the seven chromosome pseudomolecules. In two chromosome pseudomolecules, the motif was present at both ends; in three, it was present at only one end. In total, we identified seven terminal arrays of this repeat (Table 1). The lengths of these arrays ranged from 228 to 8005 bp (mean 4125 bp; sample standard deviation 2923 bp). Six arrays exceeded 3000 bp. In chromosome pseudomolecules OY986040.1 and OY986044.1, the pentanucleotide motifs on opposite sides of each had a reverse-complementary structure and orientation.

The best candidate for telomeric motif in *N. capitata* was the pentanucleotide (TTAGG)*n*; it was found in the ends of four chromosome pseudomolecules (out of eight) at one end of each. In total, we identified four terminal arrays of this repeat (Table 2)—three at 3′ ends and one at a 5′ end (reverse complement (CCTAA)*n*). Array lengths ranged from 2316 to 3808 bp (mean 2747.5 bp; sample SD 710.6 bp).

In the chromosome-level genome assemblies of both *N. flava* and *N. capitata*, no retrotransposons from the TRAS and SART families were detected. Nucleotide variation increased internally from the chromosome termini, consistent with telomeric sequence degradation patterns.

## 4. Discussion

The family Chrysopidae is widespread and species-rich, with more than 1400 described species across about 80 genera and three subfamilies: Nothochrysinae, Apochrysinae, and Chrysopinae [21]. Chrysopinae are cosmopolitan (and contain ca. 97% of the species); the Apochrysinae are restricted to tropical areas in Africa, Asia, Australia, and the Americas; and the Nothochrysinae are widespread across Europe, Australia, southern Africa, South America, and western North America [30]. Owing to their predation on arthropod pests, some Chrysopidae species are used as biological control agents in diverse agroecosystems [31]. Among the species of Chrysopidae, there are predators of agricultural pests, including spider mites (Acari: Tetranychidae), whiteflies (Hemiptera: Aleyrodidae), aphids (Hemiptera: Aphididae), mealybugs (Hemiptera: Pseudococcidae), and beetles (Coleoptera) [32,33,34]. Despite their key role as predators of agricultural pests, Chrysopidae are understudied with respect to telomeric sequence content. We identified telomeric motifs in two Chrysopidae species for the first time: (TTAGG)*n* in *N. capitata* (Nothochrysinae) and (TTGGG)*n* in *N. flava* (Chrysopinae). While previous studies documented the absence of (TTAGG)*n* in *C. carnea* [7] and *C. claveri* [24], our findings indicate that chrysopids possess alternative telomeric sequences. Notably, the (TTGGG)*n* motif represents the first such discovery in Neuroptera, suggesting greater telomeric diversity in this order than previously recognized.

The motif TTGGG has been documented as an alternative telomeric sequence in several insect groups, including beetles (Coleoptera), where it was found in *Apoderus coryli* (Linnaeus, 1758) (Attelabidae); Odonata, in the white-legged damselfly *Platycnemis pennipes* (Pallas, 1771) (Platycnemididae); and in Hymenoptera, in *Macropis europaea* Warncke, 1973 (Melittidae), where the motifs TTGGG and TTAGG are mixed, but the TTGGG motif prevailed—the TTAGG motif almost never occurs twice in a row, while TTGGG occurs in tandem arrays up to 13 repeats in length [25]. This pattern indicates that TTGGG can functionally replace the canonical TTAGG motif in telomere maintenance. Because the TTAGG to TTGGG shift requires only a single substitution and TTGGG occurs in distantly related insect orders (Coleoptera, Odonata, Hymenoptera, and now Neuroptera), the motif has most likely arisen multiple times independently.

The phylogenetic relationships among the three Chrysopidae subfamilies remain unresolved, with different studies supporting different topologies. The earliest hypothesis, scenario (1), placed Nothochrysinae as sister to Apochrysinae + Chrysopinae [30,35], but this scenario is now largely abandoned. Subsequently, nuclear and mitochondrial gene analyses supported scenario (2)—Apochrysinae as sister to Nothochrysinae + Chrysopinae [36,37]. However, more recent molecular studies, including both nuclear datasets and mitochondrial genome data, have predominantly supported a different arrangement, scenario (3), where Chrysopinae is sister to Apochrysinae + Nothochrysinae [38,39,40,41]. The picture becomes even more complex with anchored phylogenomics, which suggests that Nothochrysinae may be paraphyletic rather than forming one of three distinct clades [42]. In a study using molecular supermatrix approach, the Bayesian inference suggested scenario (2), while the tree of maximum likelihood supported scenario (3) [43]. A study combining both molecular and morphological data supports scenario (2) [44].

The distribution of (TTAGG)*n* in Nothochrysinae and (TTGGG)*n* in Chrysopinae could be more parsimoniously explained by topologies that group these subfamilies together, such as scenario (2), with the Apochrysinae + (Nothochrysinae + Chrysopinae) arrangement [36,37,44], as this would require fewer independent motif transition events. However, across insect groups, given telomeric repeat sequences occur independently in different lineages (homoplasy) [25], limiting their reliability as phylogenetically informative characters. The telomeric motif in Apochrysinae has not yet been characterized, and we lack evidence that repeat sequences are conserved within Chrysopidae lineages. Therefore, using current telomere data to distinguish among competing hypotheses of subfamily relationships would be premature. Broader taxonomic sampling across Chrysopidae, combined with independent verification methods such as fluorescence in situ hybridization (FISH) or long-read sequencing of chromosome termini, is needed to establish whether specific repeat motifs are consistently maintained within clades. Only after clade-specific conservation has been demonstrated should the observed telomeric sequences—(TTAGG)*n* in Nothochrysinae and (TTGGG)*n* in Chrysopinae species—be used to evaluate alternative phylogenetic trees based on the principle of minimizing evolutionary changes.

In most eukaryotes, a ribonucleoprotein reverse transcriptase enzyme (telomerase) is involved in telomere length maintenance. This specialized reverse transcriptase (TERT) uses an internal RNA template molecule (TR) to add short, simple repeats to chromosome ends [45]. Telomerase RNA (TR) has been scarcely studied in insects. The main exception is Hymenoptera, where a comprehensive study showed a switch to plant/ciliate-like TR biogenesis [16], which contrasts with TRs in other animals and fungi. The hymenopteran TR has multiple stem-loops positioned 3′ of the template and 5′ of the pseudoknot, which may help explain the unusual diversity of telomeric repeats reported in this order [11,25]. A similar mechanism may operate in Chrysopidae, potentially accounting for the reported telomeric sequence diversity in the family. This can be tested by studies on TRs across Chrysopidae using the approach applied by Fajkus et al. [16]. A similar notion has been previously inferred for the telomeric sequence diversity in Heteroptera [46].

The telomeric motifs identified here can be used for developing additional fluorescence in situ hybridization (FISH) probes to study/understand chromosomal organization, karyotype evolution, and genome stability in green lacewings.

## 5. Conclusions

The occurrence of (TTAGG)*n* in *N. capitata* contrasts with the reported absence of this motif in other Chrysopidae. The discovery of (TTGGG)*n* in *N. flava* represents the first documentation of this alternative pentanucleotide motif in Neuroptera, expanding the known telomeric sequence diversity within the order. The coexistence of both ancestral and alternative telomeric motifs in Chrysopidae suggests that other Neuroptera families may also harbor diverse telomeric sequences, indicating that broader surveys across the order are needed.

## Figures and Tables

**Table 1:** Best candidate for telomeric motif in *N. flava*: occurrences of (TTGGG)*n* at the ends of the seven assembled chromosome pseudomolecules. Arrays at 5′ ends are (CCCAA)*n* (reverse complement); arrays at 3′ ends are (TTGGG)*n*.

GenBank Accession	5′	3′	Chromosome Pseudomolecules Size (bp)
OY986040.1	1–8005	150,122,476–150,126,493	150,126,493
OY986041.1	no short repeat array	142,391,635–142,396,179	142,396,181
OY986042.1	1–3300	no short repeat array	119,373,907
OY986043.1	no short repeat array	100,876,893–100,877,120	100,877,120
OY986044.1	1–7554	88,716,705–88,717,929	88,717,929
OY986045.1	no short repeat array	no short repeat array	73,119,431
OY986046.1	no short repeat array	no short repeat array	41,815,626

**Table 2:** Best candidate for telomeric motif in *N. capitata*: (TTAGG)*n*, occurrence at the ends of the eight assembled chromosome pseudomolecules. Arrays at 5′ ends are (CCTAA)*n* (reverse complement); arrays at 3′ ends are (TTAGG)*n*.

GenBank Accession	5′	3′	Chromosome Pseudomolecules Size (bp)
OZ251087.1	no short repeat array	123,553,250–123,555,627	123,555,627
OZ251088.1	no short repeat array	115,084,180–115,086,667	115,086,667
OZ251089.1	1–2316	no short repeat array	93,318,919
OZ251090.1	no short repeat array	no short repeat array	61,893,665
OZ251091.1	no short repeat array	54,814,880–54,818,687	54,818,687
OZ251092.1	no short repeat array	no short repeat array	53,992,875
OZ251093.1	no short repeat array	no short repeat array	8,187,640
OZ251094.1	no short repeat array	no short repeat array	24,839,040

## Data Availability

The original contributions presented in this study are included in the article. Further inquiries can be directed at the corresponding author.
